# Preoperative assessment of coronary stenosis through ordinary chest CT for patients scheduled total hip or knee arthroplasty

**DOI:** 10.3389/fcvm.2025.1582704

**Published:** 2025-07-01

**Authors:** Yi Li, Yang Wang, Ting Ma, Xin Duan, Haibo Si

**Affiliations:** ^1^ Department of Orthopaedic Surgery and Orthopedic Research Institute, West China Hospital, Sichuan University, Chengdu, China; ^2^ Orthopaedics Department, The Sichuan Modern Hospital, Chengdu, China; ^3^ Anesthesia and Surgery Center, West China Hospital, Sichuan University, Chengdu, China

**Keywords:** coronary artery calcium, arthroplasty, coronary stenosis, ordinary chest CT, coronary angiography

## Abstract

**Background:**

Coronary artery calcium (CAC) is one of the main factors causing coronary stenosis and is often identified on ordinary chest computed tomography (CT). This study aims to explore the effectiveness of utilizing routine chest CT for preoperative coronary stenosis assessment in patients scheduled total hip or knee arthroplasty.

**Materials and methods:**

Between July 2020 and July 2024, a retrospective analysis was conducted, and a total of 293 patients who intended to perform total hip or knee arthroplasty were included from 12,150 chest CT scans. Coronary artery calcium Score (CACS) was used to evaluate coronary stenosis based on the preoperative ordinary chest CT. Correlation between CACS and the degree of coronary artery stenosis determined via the coronary angiography (CAG) and the predictive ability of CACS for coronary artery stenosis ≥50% were analyzed.

**Results:**

The number of patients with CACS scores ranging from 0 to 499 was the largest, with 139 patients (47.44%). There were 88 patients (30.03%) with coronary artery stenosis ≥50%, 40 patients (13.65%) with ≥70%, and left anterior descending artery stenosis was the most common, with a total of 72 patients (24.57%). A strong correlation (*R* = 0.891, *p* < 0.001) between the degree of coronary stenosis and CACS was observed. A CACS threshold of ≥1,500 demonstrated a specificity of 100% and a positive predictive value (PPV) of 100% for coronary stenosis of ≥50%.

**Conclusion:**

Ordinary chest CT is highly effective in evaluating the risk of coronary artery stenosis before total hip or knee arthroplasty, introducing a novel approach to facilitate our surgical decision-making.

## 1 Introduction

It is well known that atherosclerotic cardiovascular disease (ASCVD) has a high prevalence and mortality rate worldwide (1, 2). In China, ASCVD alone caused 2.4 million deaths in 2016, accounting for approximately 25% of all mortality cases (3). Coronary artery calcium (CAC) is a component of atherosclerosis (4). Investigators have noted a close correlation between calcification and severity of clinical coronary disease. Numerous studies have shown a significant association between the presence and severity of CAC and future cardiovascular disease risk (5, 6). CAC detection is one of the most sensitive, reliable, and repeatable non-invasive methods to identify subclinical atherosclerosis (7). Imaging can be used to non-invasively quantify atherosclerosis, primarily through ECG-gated computed tomography (CT) to quantify CAC. In recent years, Coronary artery calcium Score (CACS) has emerged to quantify CAC and predict the presence of coronary artery stenosis (8).

The Agatston score, the most commonly used calcium quantification method on CT, relies on software to multiply the lesion area of CAC by a density weighting factor (DWF) that is derived from the maximal CT attenuation within a given calcified lesion (7). The score for all lesions in all coronary arteries is then summed, irrespective of location or coronary distribution, to determine the total Agatston score (9, 10). The CACS is more predictive of cardiovascular events than traditional risk scores (5, 11, 12). The severity of coronary artery stenosis increases with the increase of CACS, and its prognosis can also be predicted through CACS (9, 13). It has been reported that the 10-year incidence of cardiovascular adverse events with CACS values of 0, >100, and >300 is 5.6%, 7.5%, and 13.1%, respectively (14, 15). Additional studies have demonstrated the predictive value of CACS for death, non-fatal myocardial infarction, and future revascularization (16, 17).

Ordinary chest CT is a standard medical imaging examination method mainly used to observe and evaluate the internal structure and organ lesions of the chest. Moreover, CAC can be detected during chest CT scans. As the parameters of chest CT scans are adjusted and technology advances, both ordinary and low-dose chest CT scans now exhibit high-density resolution. This improvement enables the more accurate identification of smaller lesions, CAC included.

When the CACS is applied to ordinary non-ECG gated chest CT, the modified Agatston score, calculated using low-dose non-gated CT, has demonstrated consistency with the Agatston score derived from ECG-gated scans in lung cancer screening patients and the general population (15, 18, 19). Evaluating CAC on ordinary non-contrast chest CT is a cost-effective and low-radiation method for quantifying coronary artery calcification, and it is increasingly being applied in clinical practice, such as lung cancer screening, preoperative evaluation, health check-ups, etc (20, 21). Although CAC can be detected in these routine examinations, radiologists rarely report CAC systematically and routinely, and clinical doctors rarely pay attention to these potential lesions (22, 23). In a prior study encompassing 207 patients diagnosed with CAC, radiologists reported that only 44% of patients were found to have CAC. Furthermore, within this cohort of CAC-diagnosed patients, the proportion of cases where the specific affected arteries were precisely identified was a mere 1% (23).

Presently, the guidelines for coronary artery revascularization define significant stenosis as non-left main lesion diameter stenosis ≥70% and left main lesion diameter stenosis ≥50% (24). In previous studies, Palumbo et al. advocated the use of CACS as a preliminary screening tool for coronary computed tomography angiography (CCTA) (25). Alshamrani employed various CACS cutoff values to evaluate coronary artery stenosis ≥50% and ≥70%, showing that when CACS ≥250, all symptomatic patients had coronary artery stenosis ≥50% (26). However, it must be acknowledged that the high critical value of CACS may lead to overestimating the degree of coronary artery stenosis (27). Currently, there is no universally accepted standard for CACS cutoff values to define different degrees of coronary stenosis, leading to variability in clinical interpretation and risk stratification. Prior studies have not explored the relationship between CACS based on ordinary chest CT and CAG, nor the optimal selection strategy for diagnosing coronary stenosis after detecting CAC on CT. Preoperative cardiovascular risk assessment for orthopedic surgery follows guidelines like the 2014 ACC/AHA Perioperative Cardiac Guidelines (28), which focus on clinical risk factors but often overlook subclinical coronary artery disease (CAD) detected incidentally on chest CT. CAC, a marker of atherosclerosis frequently seen on chest CT, is rarely integrated into risk stratification. This study links CAC severity to obstructive stenosis (≥50%) to inform improved pre-op CAD screening in arthroplasty patients. This study employs CAG to diagnose coronary artery stenosis and the patient population comprises those who had been detected with CAC by ordinary chest CT before total hip or knee arthroplasty. The aim is to explore the effectiveness of ordinary chest CT for preoperative coronary stenosis assessment in these patients.

## 2 Materials and methods

### 2.1 Study design

This retrospective study has been approved by the Bioethics Review Committee of West China Hospital, Sichuan University. All research data was retrieved from the medical record system. Since anonymous patient data was used in the study without any medical intervention measures, the requirement for informed consent was waived. This Retrospective Cohort Study was conducted using the STROCSS 2019 Guideline (29).

### 2.2 Patient data

**Figure 1:**
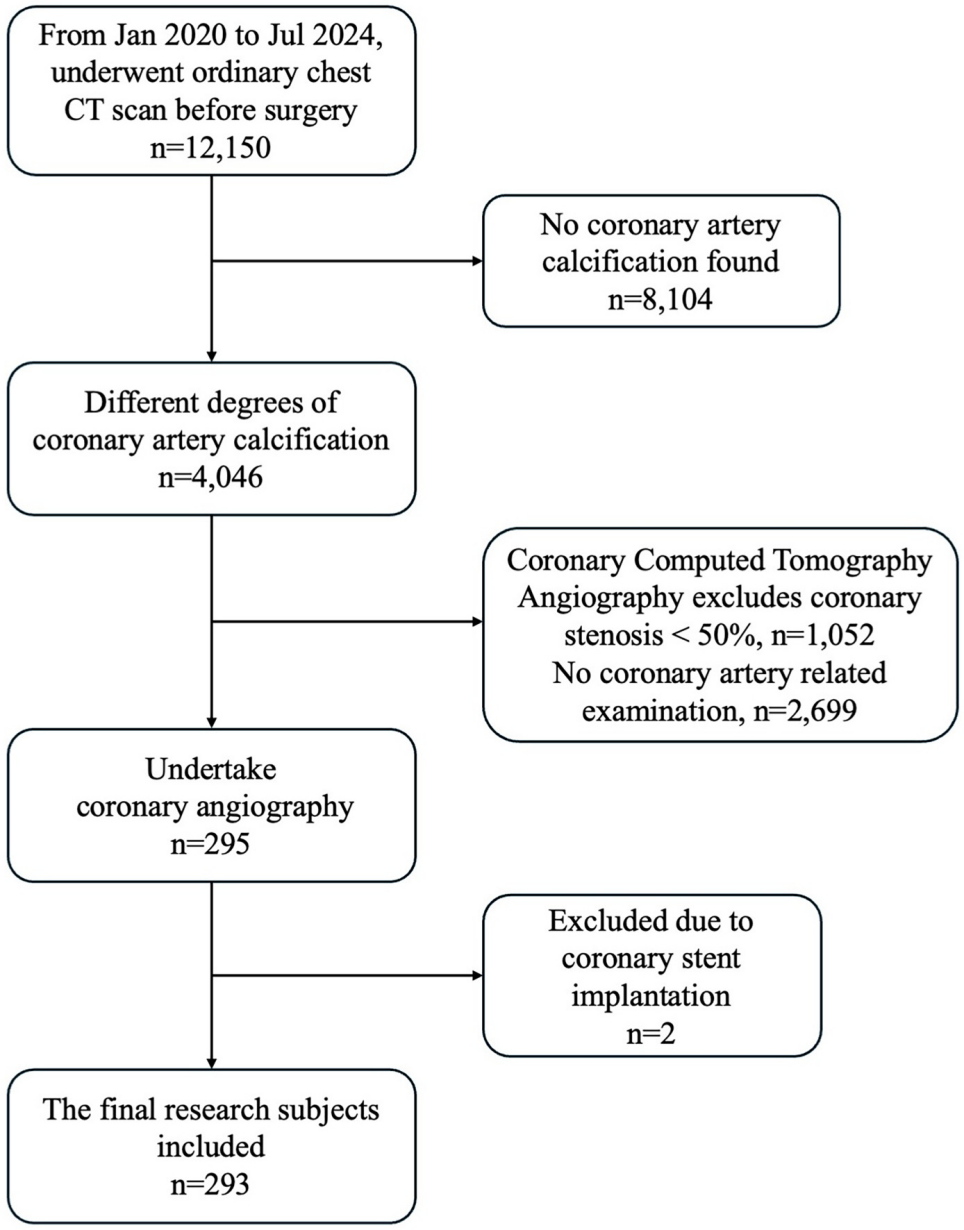
Flow diagram of patient selection process.

This retrospective study focused on patients who had been intending to undergo total hip or knee arthroplasty in our joint surgery department between July 2020 and July 2024. 12,150 patients underwent ordinary chest CT before total hip or knee arthroplasty. Among the total number of patients included in the study, no CAC was found in 8,104 patients (66.70%) and 4,046 (33.30%) were found to have varying degrees of CAC on chest CT. Patients were referred for further evaluation based on a multidisciplinary decision involving cardiologists and radiologists. The decision to proceed with CCTA or CAG was guided by: clinical symptoms (e.g., angina, dyspnea) and preferences, comorbidities and CAC severity on ordinary chest CT. 2,699 (22.21%) were excluded for not undergoing either CCTA or CAG primarily due to low clinical suspicion of Coronary Artery Disease. Additionally, 1,052 (8.66%) patients who underwent CCTA were excluded because the degree of coronary artery stenosis in these patients was <50%. 295 (2.43%) patients underwent CAG, of which two patients with a previously implanted coronary artery stent were excluded due to the potential interference of the stent with the evaluation of the current study. Consequently, a total of 293 patients were included in the final analysis (Figure 1).

The study population consisted of patients presenting with either symptomatic, including angina, dyspnea, syncope, and palpitation, or asymptomatic patients. The exclusion criteria were as follows: (1) Patients who had undergone percutaneous coronary intervention (PCI) and coronary artery bypass grafting surgery prior to admission; (2) Patients with incomplete key clinical data (such as missing preoperative imaging results). Baseline clinical data, including demographic characteristics, body mass index (BMI), laboratory tests, past medical history (including chronic diseases, medications, etc.) and surgical history, as well as the results of CACS and CAG, were collected. All diseases included in this study are defined by using the 10th revision of the International Classification of Diseases (ICD-10) diagnostic codes. The results of CACS were evaluated by three experienced radiologists blinded to the CAG results. All three cardiologists with cardiology specialist certification and over 10 years of experience in interventional treatment of ASCVD performed CAG and evaluated coronary artery stenosis severity in a blinded manner without prior knowledge of CACS results. Stenosis was graded into four categorical levels according to the 2011 ACCF/AHA/SCAI Guidelines: mild (0%–24%), slight-moderate (25%–49%), moderate (50%–69%), and severe (≥70%) (30). All doctors were blinded to each other's evaluation results. For statistical analysis, categorical grades were converted to numerical midpoints (12%, 37%, 60%, 85%), and the average of the three observers' scores was used. In cases of disagreements exceeding two categories, a fourth senior cardiologist arbitrated to reach consensus. Retrospective analysis of inter-observer agreement in a subset of 50 cases showed an intraclass correlation coefficient (ICC) of 0.82 (95% CI: 0.71–0.89) with a standard deviation (SD) of 8.7%, indicating excellent consistency across evaluations.

### 2.3 Ordinary chest CT scan and CACS

The Ordinary chest CT examinations were performed using Siemens CT scanners (SOMATOM Definition, Siemens Medical Solutions, Forchheim, Germany; and SOMATOM Definition FLASH, Siemens Medical Solutions, Forchheim, Germany) or a Revolution CT scanner (GE Healthcare, Waukesha, WI, USA) with patients in the supine position. The scan covered the area from the lung's apex down to the lower boundary of the costophrenic angle, with a slice thickness of 1 mm. No contrast agents or drugs were injected during the scanning period. Subsequently, the initial data set was reconstructed upon completion of the scan, and images were transferred to image-processing workstations for image analysis. The CACS was determined using Agatston's algorithm (10, 31). CACS was performed utilizing commercially available calcium scoring software (PHILIPS), which was used to identify and score any calcium in the four main coronary arteries based on established minimum attenuation values. Finally, the Agatston score was generated by summing the scores of all lesions, which were derived by multiplying the lesion area CAC by DWF.

### 2.4 Coronary angiography (CAG)

The cardiologists, blinded to the CACS results, performed a right radial artery puncture using Seldinger's technique and inserted a 6Fr sheath. Heparin (3,000 μ) was administered with a 6Fr Heartrail angiography catheter before coronary angiography. Subsequently, the severity of stenosis in the left main coronary artery (LM), left anterior descending artery (LAD), left circumflex artery (LCX), and right coronary artery (RCA) was evaluated. Finally, the cardiologists provided relevant reports and images based on the operation.

### 2.5 Statistical analysis

All statistical analyses were performed using SPSS software (version 27.0, SPSS Inc., USA). Characteristics of the study population were presented as mean ± SD (standard deviation) for continuous variables that followed a normal distribution, while categorical variables were expressed in percentages. Additionally, continuous variables that were not normally distributed were described using the median and interquartile range. Differences in categorical variables were assessed using a Chi-square test. Then, the correlation between CACS and coronary stenosis severity was assessed using Pearson's correlation coefficient, with stenosis categories converted to numerical midpoints. Finally, the severity and location of coronary stenosis were evaluated by CAG. A receiver operating characteristic (ROC) curve analysis was performed, including calculations of specificity, sensitivity, positive predictive value (PPV), negative predictive value (NPV), and area under the ROC curve (AUC) for evaluating the ability of CACS to predict coronary stenosis. The Youden index was used to identify the best cutoff point. The diagnostic performance of different CACS cutoff values based on routine chest CT was assessed to detect coronary stenosis with degrees of ≥50% and ≥70%. Moreover, the significance level was set at a bilateral *P*-value threshold of <0.05.

## 3 Results

### 3.1 Patient characteristics

Among 293 patients, 87 were male and 206 were female. Ages ranged from 42 to 89, with a mean of 69.31 ± 7.97 years. Of them, 92 had total hip arthroplasty (THA), 193 had total knee arthroplasty (TKA), and 8 didn't proceed with the surgery. The median CACS was 632 (from 12 to 2016, with an interquartile range of 230.5–898.5). 205 (69.97%) patients did not have significant coronary artery stenosis (<50%), while 88 (30.0%) exhibited mild (50%–69%, *n* = 48) to severe (≥70%, *n* = 40) coronary stenosis (Table 1).

**Table 1:** Clinical characteristics of the study population.

Clinical characteristics	Value
Age, years	69.31 ± 7.97
Female	206 (70.3%)
THA	92 (31.4%)
TKA	193 (59.0%)
BMI (kg/m^2^)	25.43 ± 3.63
Systolic blood pressure (mmHg)	140.00 (127.00–150.50)
Diastolic blood pressure (mmHg)	85.29 ± 12.03
Hypertension	154 (52.6%)
Heart rate (times/min)	77 ± 11
EF (%)	67 (63–15071)
Coronary artery disease	46 (15.7%)
Diabetes mellitus	43 (14.7%)
History of smoking	30 (10.2%)
Osteoporosis	98 (33.4%)
Renal insufficiency	11 (3.8%)
Autoimmune disease	14 (4.9%)
COPD	9 (3.1%)
Cr (μmol/L)	65 (56–77)
Cholesterol (mmol/L)	4.64 ± 1.12
HDL (mmol/L)	1.32 (1.11 ± 1.57)
LDL (mmol/L)	2.75 ± 0.96
TG (mmol/L)	2.25 (2.13–2.34)
Calcium (mmol/L)	2.24 ± 0.13
CACS	
0–499	139 (47.44%)
500–999	97 (33.11%)
1,000–1,499	37 (12.63%)
≥1,500	20 (6.83%)
The region with the most pronounced coronary artery stenosis	
≤24%	119 (40.61%)
25%–49%	86 (29.35%)
50%–69%	48 (16.38%)
≥70%	40 (13.65%)

Values are mean ± SD, *n* (%), or median (interquartile range). BMI, body mass index; EF, ejection fraction; COPD, chronic obstructive pulmonary disease; Cr, Creatinine; HDL, high-density lipoprotein cholesterol; LDL, low-density lipoprotein cholesterol; TG, triglyceride; CACS, coronary artery calcium score.

### 3.2 Predictive value of CACS in coronary stenosis evaluation

**Figure 2:**
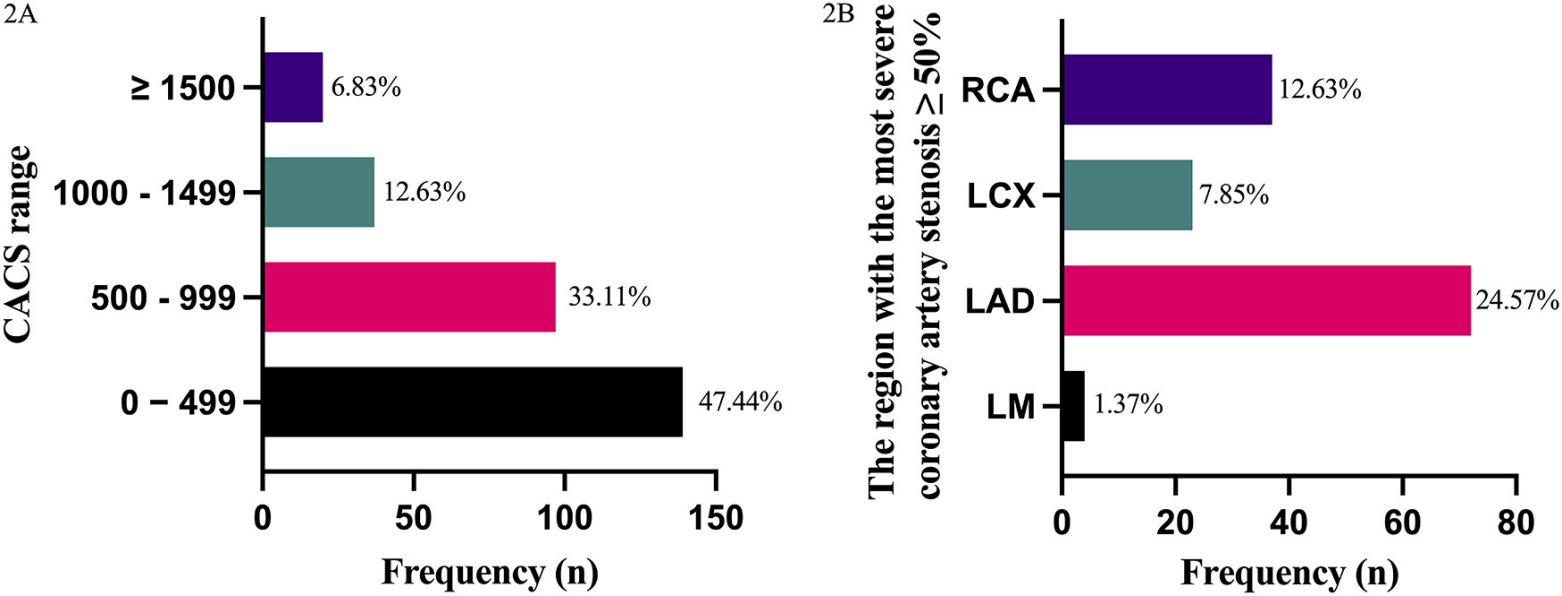
Predictive value of CACS in coronary stenosis evaluation. **(A)** Frequency of coronary artery calcium scores (CACS) categories in 293 patients scheduled for arthroplasty. *X*-axis: CACS groups (0–499, 500–999, 1,000–1,499, ≥1,500). *Y*-axis: Number of patients. **(B)** Frequency of severe coronary stenosis (≥50%) by artery location in coronary angiography (CAG). *X*-axis: Coronary artery segments (LM, left main coronary artery; LAD, left anterior descending coronary artery; LCX, left circumflex coronary artery; RCA, right coronary artery). *Y*-axis: Number of patients.

The distribution of CACS within the cohort was shown in Figure 2A, with the highest number of patients (*n* = 139, 47.44%) having scores between 0 and 499. The region of the most severe coronary artery stenosis ≥50% in CAG was shown in Figure 2B, where LAD stenosis was found to be the most common (*n* = 72, 24.57%). Meanwhile,a strong correlation between the coronary stenosis degree and CACS was also observed (Pearson correlation *R* = 0.891, *p* < 0.001).

**Figure 3:**
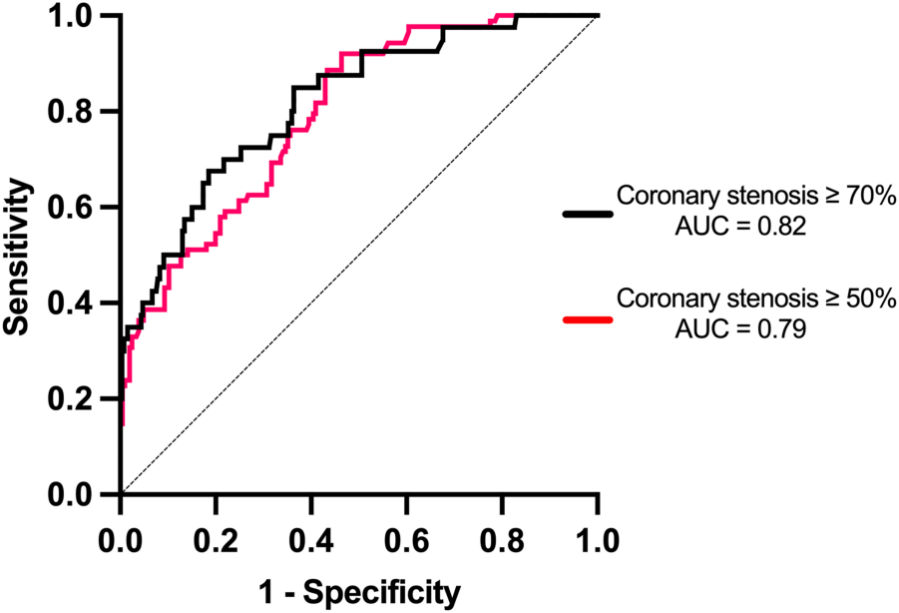
Receiver operating characteristic (ROC) curves evaluating the performance of CACS in detecting coronary stenosis of ≥50% and ≥70% on coronary angiography (CAG). For ≥50% stenosis, the optimal CACS cutoff was 350.5, yielding a sensitivity of 92.0%, specificity of 53.7%, PPV of 42.9%, NPV of 93.3%, and accuracy of 60.8% (AUC = 0.79, 95% CI: 0.73–0.85). For ≥70% stenosis, the optimal cutoff was 856.5, with sensitivity of 67.5%, specificity of 81.4%, PPV of 17.3%, NPV of 87.6%, and accuracy of 69.6% (AUC = 0.82, 95% CI: 0.75–0.89). Dashed line represents the reference line (AUC = 0.5).

Based on the ROC curve analysis, for the detection of coronary stenosis with a severity of ≥50%, the optimal CACS cutoff point was 350.5. At this value, the sensitivity reached 92.0%, the specificity was 53.7%, the positive predictive value (PPV) was 42.9%, the negative predictive value (NPV) was 93.3%, and the accuracy was 60.8% (AUC = 0.79). For coronary stenosis ≥70%, the optimal CACS cutoff point was 856.5, resulting in a sensitivity of 67.5%, a specificity of 81.4%, a PPV of 17.3%, an NPV of 87.6%, and an accuracy of 69.6% (AUC = 0.82) (Figure 3). The diagnostic efficacy of various CACS cutoff values for coronary artery stenosis ≥50% was presented in Table 2. Specifically, when the CACS cutoff was set at ≥1,500, a sensitivity of 22.3% was exhibited, a specificity of 100% was shown, a PPV of 100% was demonstrated, an NPV of 75.1% was revealed, and a diagnostic accuracy of 76.8% was achieved. In contrast, when CACS ≤143, coronary stenosis ≥50% was not found in any patients.

**Table 2:** Different cutoff values of CACS to detect coronary stenosis ≥50%.

CACS cutoff value	Degree of stenosis	Number of patients (%)	Sensitivity (%)	Specificity (%)	PPV (%)	NPV (%)	Accuracy (%)
≥300	≥50%	81 (27.6%)	92.0	47.3	42.9	93.3	60.8
≥600	≥50%	65 (22.2%)	73.9	64.4	47.1	85.2	67.2
≥1,000	≥50%	38 (13.0%)	43.2	90.2	65.5	78.7	76.1
≥1,300	≥50%	28 (9.6%)	31.8	97.1	82.4	76.8	77.5
≥1,500	≥50%	20 (6.8%)	22.3	100	100	75.1	76.8

## 4 Discussion

Total joint arthroplasty is an excellent solution for end-stage joint diseases, and its prevalence has increased yearly (32). The future demands for primary total hip and knee arthroplasties are estimated to grow by nearly 2-fold and 7-fold, respectively, with middle-aged and elderly patients accounting for the vast majority (33). The study highlights the clinical utility of chest CT-derived CACS in orthopedic preoperative screening for total hip/knee arthroplasty. Routine chest CT, primarily for pulmonary assessment, can secondarily identify unsuspected CAD via calcification detection. A high CACS (e.g., ≥1,500) correlates strongly with significant stenosis (≥50%), enabling risk stratification to guide further cardiac evaluation (e.g., CCTA/CAG) and optimize perioperative care. We found that CACS is ubiquitous in patients undergoing THA and TKA. Most patients exhibit a score between 0 and 499, accounting for 47.44%. Among patients with CAC, a total of 88 patients were found to have significant stenosis of the coronary arteries (≥50%), with LAD stenosis being the most common. Among the 88 patients, 72 had significant stenosis of LAD, accounting for 24.57%. In general, the results of this study indicated a strong correlation between CACS and the degree of coronary artery stenosis (Pearson correlation *R* = 0.891, *p* < 0.001), which is consistent with previous research findings that the CACS based on ordinary chest is a reliable cardiovascular risk prediction method (3, 18, 34).

We conducted an ROC curve analysis to evaluate the predictive ability of CACS for coronary artery stenosis, revealing that the AUC of coronary artery stenosis ≥70% was higher than that of coronary artery stenosis ≥50%. Besides, this study assessed multiple CACS cutoff values for diagnosing coronary stenosis and investigated the association between CACS and the presence of coronary stenosis. Since the evaluation of coronary artery stenosis is crucial for surgical decision-making in patients undergoing procedures like THA and TKA (where the presence of coronary artery disease may impact the surgical risk and outcome), accordingly, we focused on exploring the relationship between CACS values and coronary artery stenosis ≥50% and ≥70% (24).

Previous studies by De Agustin et al. showed that in patients with chest pain and CACS ≥400, the specificity and PPV for detecting coronary artery stenosis ≥70% were 93.5% and 85.8%, respectively (35). Therefore, it is recommended that patients with chest pain symptoms and CACS ≥400 should avoid undergoing CCTA examination and instead undergo CAG. Previous studies also have indicated a positive correlation between higher CACS and an increased likelihood of coronary stenosis (36–38). Since CCTA may offer little extra value for patients with a high CACS-indicating a high likelihood of ≥50% coronary stenosis, these patients can make an informed decision about foregoing CCTA and instead choose early CAG and decide whether to undergo percutaneous coronary revascularization based on the results. The ≥1,500 threshold was optimized for maximal specificity and PPV in our population, but its generalizability may be limited in cohorts with different prevalence of obstructive disease or imaging protocols. External validation in diverse datasets is essential to confirm its robustness.

The application of CCTA and the gold-standard CAG in coronary artery examination requires further study. Some patients skipped CAG as CCTA showed no significant stenosis. CCTA has advantages. Its high specificity helps rule out non-obvious stenosis in the initial screening, reducing unnecessary follow-up tests (39, 40). However, a high false-positive rate may lead to needless further checks (41). As a non-therapeutic tool of diagnosis, CCTA, when it reveals severe stenosis, indicates that patients still need CAG. This not only increases costs but also raises the risks of complications such as renal problems, thyroid-related issues, and hypersensitivity reactions caused by repeated contrast agent use (42, 43).

Our study also found that when the patient's CACS ≤143, there was no significant coronary artery stenosis. Hence, we advise that when the preoperative chest CT shows CACS ≤143, usually no special treatment or additional test needs to be performed unless the patient exhibits obvious chest pain symptoms or a long-term history of coronary heart disease. However, CACS cannot visualize coronary stenosis caused by non-calcified plaque. In patients with CACS = 0, most plaques were predominantly non-calcified (44). It is worth noting that the early stages of coronary atherosclerosis do not exhibit any calcification. Among the 12 patients with CACS = 0 and coronary artery stenosis ≥50%, non-calcified plaques were found in 10 cases (45). Therefore, we deem that for asymptomatic or chest pain patients, if the patient has multiple risk factors, such as age, family history, smoking, diabetes, hypertension, and abnormal blood lipid levels, even if ordinary chest CT shows no CAC, medical service providers can also consider CCTA as a non-invasive imaging method to assess the health status of coronary arteries preliminarily. For patients with significant stenosis on CCTA examination, early CAG to clarify the degree and location of the lesion and timely treatment is extremely necessary. In recent years, coronary magnetic resonance angiography (MRA), with its non-invasive nature, absence of ionizing radiation, option of non-contrast use, and low susceptibility to vascular calcification, has emerged as a promising new method for ASCVD screening and is nearly ready for clinical application (46–48). However, its complex imaging technique and long acquisition time currently impede large-scale implementation. Despite these challenges, the future of coronary MRA is bright.

There were some limitations in this study. Firstly, non-ECG-gated chest CT is prone to artifacts such as motion and respiratory artifacts, which complicate the calculation of CACS and may affect accuracy. In future studies, it is recommended to integrate ECG gating or phase-reconstruction algorithms, such as using motion-corrected deep learning models, to mitigate errors caused by artifacts. Secondly, a key limitation of our study is the use of the Agatston algorithm to calculate CACS. This method fails to account for calcium density within plaques, instead assigning higher scores to denser calcifications. Paradoxically, prior research shows that higher calcium density within a given plaque volume correlates inversely with cardiovascular events. As a result, CACS may overstate the risk of dense plaques, potentially causing misestimation of cardiovascular risk. Thirdly, this study did not explore the relationship between non-CAC and coronary stenosis. Additionally, the total CACS did not offer a distinct evaluation of calcification in individual coronary vessels, especially the situation of single-vessel involvement (such as the degree of CAC and CACS in single vessels). This will remind doctors of the presence of severe coronary artery disease. Another limitation of this study is that the cutoff values for predicting coronary artery stenosis were not adjusted for age and gender. As a result, the established cutoff values may not accurately reflect the risk of coronary artery stenosis across different age and gender subgroups. This study focused on patients with moderate-to-severe coronary stenosis, excluding those with <50% stenosis after CCTA. This exclusion criterion may overestimate the predicted value of CACS. Moreover, the lack of standardized protocols and scoring criteria for coronary calcium scoring, along with the potential for missed detection or underestimation of mild calcifications, remains a limitation. Future research should leverage advanced software algorithms (e.g., deep learning, image fusion) and standardized scanning/post-processing procedures to enhance the reliability of chest CT-based calcium scores comparable to those from coronary CT angiography.

Ultimately, these improvements will significantly enhance the precision and clinical relevance of risk predictions grounded in CACS. This cross-disciplinary approach aligns with trends in shared decision-making but requires validation in larger cohorts and consideration of radiation/cost for broader use. Future studies should assess whether CACS screening reduces perioperative cardiac events to support its integration into standardized pre-op protocols.

## 5 Conclusion

The application of CACS derived from ordinary chest CT proves advantageous for evaluating coronary stenosis risk before total hip and knee arthroplasties. High CACS cutoff values exhibit high specificity and PPV in diagnosing coronary stenosis ≥50% and ≥70%. When preoperative CACS ≥1,500, we suggest patients skip CCTA and directly have CAG for a definite diagnosis and possible concurrent treatment. In short, preoperative coronary stenosis risk assessment based on ordinary chest CT might represent a novel approach that assists us in making surgical decisions. However, further high-quality research is essential to refine criteria.

## Data Availability

The raw data supporting the conclusions of this article will be made available by the authors, without undue reservation.
