# Supplementary material for: Preoperative assessment of coronary stenosis through ordinary chest CT for patients scheduled total hip or knee arthroplasty
Source: Front Cardiovasc Med. 2025 Jul 1;12:1582704. doi: 10.3389/fcvm.2025.1582704 (PMC12260461; doi:10.3389/fcvm.2025.1582704)
Supplement: Supplementary file 1 [file Table1.doc]

| **The STROCSS Guideline** | | |
| --- | --- | --- |
| **Item**  **no.** | **Item description** | **Page No.** |
| **TITLE** | | |
| 1 | Title:  - The word cohort or cross-sectional or case-controlled is included  - The area of focus is described (e.g. disease, exposure/intervention, outcome)  - Key elements of study design are stated (e.g. retrospective or prospective) | 1 |
| **ABSTRACT** | | |
| 2a | Introduction: the following points are briefly described  - Background  - Scientific Rationale for this study | 1 |
| 2b | Methods: the following areas are briefly described  - Study design (cohort, retro-/prospective, single/multi-centred)  - Patient populations and/or groups, including control group, if applicable  - Interventions (type, operators, recipients, timeframes)  - Outcome measures | 1 |
| 2c | Results: the following areas are briefly described  - Summary data (with statistical relevance) with qualitative descriptions, where appropriate | 1 |
| 2d | Conclusion: the following areas are briefly described  - Key conclusions  - Implications to practice  - Direction of and need for future research | 1 |
| **INTRODUCTION** | | |
| 3 | Introduction: the following areas are described in full  - Relevant background and scientific rationale  - Aims and objectives  - Research question and hypotheses, where appropriate | 1-2 |
| **METHODS** | | |
| 4a | Registration and ethics  - Research Registry number is stated, in accordance with the declaration of Helsinki*  - All studies (including retrospective) should be registered before submission  *"*Every research study involving human subjects must be registered in a*  *publicly accessible database before recruitment of the first subject*" (this can be obtained from; ResearchRegistry.com or ClinicalTrials.gov or ISRCTN) | 3 |
| 4b | Ethical Approval: the following  areas are described in full  - Necessity for ethical approval  - Ethical approval, with relevant judgement reference from ethics committees  - Where ethics was unnecessary, reasons are provided | 3 |
| 4c | Protocol: the following areas are described comprehensively  - Protocol (*a priori* or otherwise) details, with access directions  - If published, journal mentioned with the reference provided | 3 |

| 4d | Patient involvement in Research  - Describe how, if at all, patients were involved in study design e.g. were they involved on the study steering committee, did they provide input  on outcome selection, etc. | 3 |
| --- | --- | --- |
| 5a | Study Design: the following areas are described comprehensively - ‘Cohort’ study is mentioned  - Design (e.g. retro-/prospective, single/multi-centred) | 3 |
| 5b | Setting: the following areas are described comprehensively  - Geographical location  - Nature of institution (e.g. academic/community, public/private)  - Dates (recruitment, exposure, follow-up, data collection) | 3 |
| 5c | Cohort Groups: the following areas are described in full  - Number of groups  - Division of intervention between groups | 3 |
| 5d | Subgroup Analysis: the following areas are described comprehensively - Planned subgroup analyses  - Methods used to examine subgroups and their interactions | 3 |
| 6a | Participants: the following areas are described comprehensively  - Eligibility criteria  - Recruitment sources  - Length and methods of follow-up | 3 |
| 6b | Recruitment: the following areas are described comprehensively - Methods of recruitment to each patient group  - Period of recruitment | 3 |
| 6c | Sample Size: the following areas are described comprehensively - Margin of error calculation  - Analysis to determine study population  - Power calculations, where appropriate | 3 |
| **Intervention and Considerations** | | |
| 7a | Pre-intervention Considerations: the following areas are described comprehensively  - Patient optimisation (pre-surgical measures)  - Pre-intervention treatment (hypothermia/-volaemia/-tension; ICU care; bleeding problems; medications) | 3-4 |
| 7b | Intervention: the following areas are described comprehensively  - Type of intervention and reasoning (e.g. pharmacological, surgical, physiotherapy, psychological)  - Aim of intervention (preventative/therapeutic)  - Concurrent treatments (antibiotics, analgaesia, anti-emetics, NBM, VTE prophylaxis)  - Manufacturer and model details where applicable | 3-4 |
| 7c | Intra-Intervention Considerations: the following areas are described comprehensively  - Administration of intervention (location, surgical details, anaesthetic, positioning, equipment needed, preparation, devices, sutures,  operative time)  - Pharmacological therapies include formulation, dosages, routes and durations  - Figures other media are used to illustrate | 3-4 |

| 7d | Operator Details: the following areas are described comprehensively - Training needed  - Learning curve for technique  - Specialisation and relevant training | 4 |
| --- | --- | --- |
| 7e | Quality Control: the following areas are described comprehensively  - Measures taken to reduce variation  - Measures taken to ensure quality and consistency in intervention delivery | 4 |
| 7f | Post-Intervention Considerations: the following areas are described comprehensively  - Post-operative instructions and care  - Follow-up measures  - Future surveillance requirements (e.g. imaging, blood tests) | 4 |
| 8 | Outcomes: the following areas are described comprehensively - Primary outcomes, including validation, where applicable  - Definitions of outcomes  - Secondary outcomes, where appropriate  - Follow-up period for outcome assessment, divided by group | 4 |
| 9 | Statistics: the following areas are described comprehensively  - Statistical tests, packages/software used, and interpretation of significance  - Confounders and their control, if known  - Analysis approach (e.g. intention to treat/per protocol)  - Sub-group analysis, if any | 4 |
| **RESULTS** | | |
| 10a | Participants: the following areas are described comprehensively  - Flow of participants (recruitment, non-participation, cross-over and withdrawal, with reasons)  - Population demographics (prognostic features, relevant socioeconomic features, and significant numerical differences) | 4-5 |
| 10b | Participant Comparison: the following areas are described comprehensively  - Table comparing demographic included  - Differences, with statistical relevance  - Any group matching, with methods | 4-5 |
| 10c | Intervention: the following areas are described comprehensively  - Changes to interventions, with rationale and diagram, if appropriate  - Learning required for interventions  - Degree of novelty for intervention | 4-5 |
| 11a | Outcomes: the following areas are described comprehensively  - Clinician-assessed and patient-reported outcomes for each group  - Relevant photographs and imaging are desirable  - Confounders to outcomes and which are adjusted | 4-5 |
| 11b | Tolerance: the following areas are described comprehensively  - Assessment of tolerance  - Loss to follow up, with reasons (percentage and fraction)  - Cross-over with explanation | 5 |
| 11c | Complications: the following areas are described comprehensively - Adverse events described  - Classified according to Clavien-Dindo classification* | 5 |

|  | - Mitigation for adverse events (blood loss, wound care, revision surgery should be specified)  *Dindo D, Demartines N, Clavien P-A. Classification of Surgical  Complications. A New Proposal with Evaluation in a Cohort of 6336 Patients and Results of a Survey. Ann Surg. 2004; 240(2): 205-213 | 5 |
| --- | --- | --- |
| 12 | Key Results: the following areas are described comprehensively - Key results, including relevant raw data  - Statistical analyses with significance | 5 |
| **DISCUSSION** | | |
| 13 | Discussion: the following areas are described comprehensively  - Conclusions and rationale  - Reference to relevant literature  - Implications to clinical practice  - Comparison to current gold standard of care  - Relevant hypothesis generation | 5-7 |
| 14 | Strengths and Limitations: the following areas are described comprehensively - Strengths of the study  - Limitations and potential impact on results  - Assessment of bias and management | 5-7 |
| 15 | Implications and Relevance: the following areas are described  comprehensively  - Relevance of findings and potential implications to clinical practice are detailed  - Future research that is needed is described, with study designs detailed | 5-7 |
| **CONCLUSION** | | |
| 16 | Conclusions:  - Key conclusions are summarised  - Key directions for future research are summarised | 7 |
| **DECLARATIONS** | | |
| 17a | Conflicts of interest  - Conflicts of interest, if any, are described | 1 |
| 17b | Funding  - Sources of funding (e.g. grant details), if any, are clearly stated | 1 |
